# A Literature Review of Economic Evaluations for a Neglected Tropical Disease: Human African Trypanosomiasis (“Sleeping Sickness”)

**DOI:** 10.1371/journal.pntd.0003397

**Published:** 2015-02-05

**Authors:** C. Simone Sutherland, Joshua Yukich, Ron Goeree, Fabrizio Tediosi

**Affiliations:** 1 Swiss Tropical and Public Health Institute, Basel, Switzerland; 2 University of Basel, Basel, Switzerland; 3 Department of Global Health Systems and Development, Tulane University School of Public Health and Tropical Medicine, New Orleans, Louisiana, United States of America; 4 Programs for Assessment of Technology in Health (PATH) Research Institute, St. Joseph’s Healthcare Hamilton, Hamilton, Ontario, Canada; 5 Department of Clinical Epidemiology & Biostatistics, McMaster University, Hamilton, Ontario, Canada; 6 Centre for Research on Health and Social Care Management (CERGAS), Università Bocconi, Milano, Italy; Foundation for Innovative New Diagnostics (FIND), SWITZERLAND

## Abstract

Human African trypanosomiasis (HAT) is a disease caused by infection with the parasite *Trypanosoma brucei gambiense* or *T. b. rhodesiense*. It is transmitted to humans via the tsetse fly. Approximately 70 million people worldwide were at risk of infection in 1995, and approximately 20,000 people across Africa are infected with HAT. The objective of this review was to identify existing economic evaluations in order to summarise cost-effective interventions to reduce, control, or eliminate the burden of HAT. The studies included in the review were compared and critically appraised in order to determine if there were existing standardised methods that could be used for economic evaluation of HAT interventions or if innovative methodological approaches are warranted. A search strategy was developed using keywords and was implemented in January 2014 in several databases. The search returned a total of 2,283 articles. After two levels of screening, a total of seven economic evaluations were included and underwent critical appraisal using the Scottish Intercollegiate Guidelines Network (SIGN) Methodology Checklist 6: Economic Evaluations. Results from the existing studies focused on the cost-effectiveness of interventions for the control and reduction of disease transmission. Modelling was a common method to forecast long-term results, and publications focused on interventions by category, such as case detection, diagnostics, drug treatments, and vector control. Most interventions were considered cost-effective based on the thresholds described; however, the current treatment, nifurtomix-eflornithine combination therapy (NECT), has not been evaluated for cost-effectiveness, and considerations for cost-effective strategies for elimination have yet to be completed. Overall, the current evidence highlights the main components that play a role in control; however, economic evaluations of HAT elimination strategies are needed to assist national decision makers, stakeholders, and key funders. These analyses would be of use, as HAT is currently being prioritized as a neglected tropical disease (NTD) to reach elimination by 2020.

## Background

Human African trypanosomiasis (HAT) is a disease caused by infection with the parasite *Trypanosoma brucei gambiense* or *T*. *b*. *rhodesiense* and is transmitted to humans via the tsetse fly. Approximately 70 million people worldwide were at risk of infection in 1995 [1], and although 7,216 cases were reported in 2012 [2], it is estimated that approximately 20,000 people across Africa are infected with HAT [2]. According to the Global Burden of Disease, recent estimates of years lived with disability (YLDs) for HAT annually range from 2,000 to 25,000 [3]. There are approximately 30 African countries affected by this disease, and it has been identified by the World Health Organization (WHO) as a neglected tropical disease (NTD) [4].

WHO describes the disease as a neurological breakdown that is caused by the trypanosome parasite in the brain, which eventually leads to a coma or death if a patient is not treated [5]. Patients are identified by self-reporting to health care centres (referred to as “passive case detection”), while active screening by trained professionals in mobile teams continues in high- and moderate-transmission areas. Active screening campaigns are carried out in remote villages, and a series of tests are used for the diagnosis of the disease. The current diagnostic algorithms for HAT include the card agglutination test for trypanosomiasis (CATT) followed by full blood assays to identify the parasite microscopically. Lumbar puncture with parasitological confirmation is then used for staging of the disease. Patients that are diagnosed with HAT are then referred to HAT treatment centres. Limited active screening is done for *T*. *b*. *rhodesiense* because there is no serological test available to facilitate easy identification. Hence, most *T*. *b*. *rhodesiense* cases are detected by clinical signs and symptoms. The subsequent diagnostic steps are similar to *T*. *b*. *gambiense* in that parasite detection is done using chancre aspirate or blood, and staging of the disease again uses cerebrospinal fluid obtained from lumbar puncture. The treatments for *T*. *b*. *gambiense* and *T*. *b*. *rhodesiense* also differ. Treatment for *T*. *b*. *gambiense* includes a 7-day intramuscular injection treatment of pentamidine for patients in stage 1 of the disease that is generally well tolerated, with minor adverse events. Nifurtimox-eflornithine combination therapy (NECT) is a 14-day in-hospital chemotherapy treatment that is required for patients suffering from stage 2 of HAT. The adverse events commonly seen in patients treated with NECT are considered to be mild to moderate in severity. For HAT *T*. *b*. *rhodesiense*, the treatment for stage 1 includes weekly intravenous injections of suramin over the course of 5 weeks [5]. Negative reactions to suramin coincide with the patient’s health status, but overall, it is a well-tolerated treatment. Stage 2 treatment for *T*. *b*. *rhodesiense* is a 10-day treatment of melarsoprol. Melarsoprol is the most toxic of the HAT treatments, leading to encephalopathic syndrome in 5% to 18% of patients treated and often resulting in death. Vector control methods for prevention of HAT *T*. *b*. *rhodesiense* are commonly used, as the disease is well-known to have an animal reservoir that contributes to transmission in both human and animal populations [5]. In regards to HAT *T*. *b*. *gambiense*, historically, vector control has not been suggested. However, evidence of an animal reservoir for *T*. *b*. *gambiense* has been discussed [6,7], and vector control was recently encouraged by WHO as an integrated strategy for HAT [5].

The year scheduled for HAT elimination is 2020 [8], and as this deadline approaches, research groups are currently developing new drug treatments and diagnostic tools [9–11] for HAT. Additionally, experts in vector control methods are also seeking interventions that would be more cost-effective and feasible for communities at risk for the disease. Even traditional teams that have gone out via trucks are now being reconsidered in combination with newer drug treatments using motorbike teams. Although some screening programs include a component of community sensitization, community involvement within control and elimination campaigns and knowledge of how this “disease awareness” is translated into behavioural changes and attitudes within affected populations need to be considered. There is now a need to evaluate not only the possibility of control and elimination for HAT but also how these new interventions and approaches may contribute to the grand scheme of such endeavours.

WHO has provided recommendations to improve certain factors likely to achieve elimination [2], and decision makers have also committed to funding the elimination of the disease [12]; yet, a clear path to the achievement of this goal is not available, nor is it clear what the most efficient pathway towards elimination would be. In addition, thus far there has been no synthesis of the current costs and effectiveness of all strategies that could intervene in the transmission of the disease. The objective of this review was to identify existing economic evaluations in order to summarise cost-effective interventions to reduce, control, or eliminate the burden of HAT. The studies included in the review were compared and critically appraised in order to determine if there were standardised methods that could be used for economic evaluations of HAT interventions or if innovative methodological approaches are warranted.

## Methods

### Literature Search Strategy

A literature search was conducted via the OvidSp interface on January 22, 2014 using keywords for HAT specific to the Medical Subject Headings (MeSH) terms required for Medical Literature Analysis and Retrieval System Online (MEDLINE) and Embase databases. An economic filter developed by Scottish Intercollegiate Guidelines Network (SIGN) was also applied. (Refer to S1 Supporting Information) The Journal Storage (JSTOR) database was also searched using the following key words: African trypanosomiasis OR trypanosom& OR “sleeping sickness” AND cost& AND economics. In addition, the following keywords were also searched in the Database of Abstracts of Reviews of Effects (DARE), National Health Service Economic Evaluation Database Health Technology Assessment (NHSEED HTA), and Cochrane databases: “African” AND “Trypanosomiasis” OR “sleeping sickness”. All citations were downloaded into Mendeley, where duplicates were identified and removed.

### Literature Screening & Inclusion/Exclusion Criteria

Screening of the articles was done in two stages. At the first level, all titles and abstracts were screened. Articles that were considered potentially relevant were then assessed at the second level, in which the full text was read. After reading the full text, articles that still met the inclusion criteria were considered. A full description of the inclusion and exclusion criteria is available in S2 Supporting Information. Data were screened on both levels according to the outline of population-intervention-comparators-outcomes-setting (PICOS) criteria, in which the population pertained to humans. Evaluations regarding strains of both HAT *T*. *b*. *gambiense* and *T*. *b*. *rhodesiense* were reviewed, although outcomes only pertaining to humans impacted by the disease were taken into consideration (no animal implications). Interventions (I) and comparators (C) included any intervention that could lead to prevention or reduction of disease in human populations (including vector control). The outcomes (O) that were considered for review were costs, consequences (life-years saved [LYS], disability-adjusted life years [DALYs], etc.), and the incremental cost-effectiveness ratio (ICER), while the setting (S) included any African country. For the purpose of this analysis, an economic evaluation was defined by the Drummond et al. definition of a “full economic evaluation,” and therefore, both costs and consequences of two or more alternatives had to be present in the analyses evaluated [13]. In cases in which an incremental analysis was not performed, articles were not excluded. Instead, if there was sufficient information in the publication to calculate the ICER, it was calculated during the review process. If there was insufficient information to calculate the ICER, it was noted in the critical appraisal that an incremental analysis was not present. No time constraints were added to the search.

### Quality Assessment and Critical Appraisal

The quality of the included studies was assessed using the SIGN Methodology Checklist 6: Economic Evaluations Version 3.0 [14], which was composed of two parts. The first portion contained questions regarding the internal and external validity of the publication. Items in the sections were assessed using answers of “Yes,” “No,” or “Can’t say.” The second portion of the checklist addressed the reviewers overall assessment of the study and also provided the reviewer with an area to judge if the article was “unacceptable,” “acceptable,” or of “high quality.” Studies that received a “Yes” on 65% or more of the questions in Section 1 were considered acceptable to the authors.

## Results

### Literature Search Results

**Fig 1:**
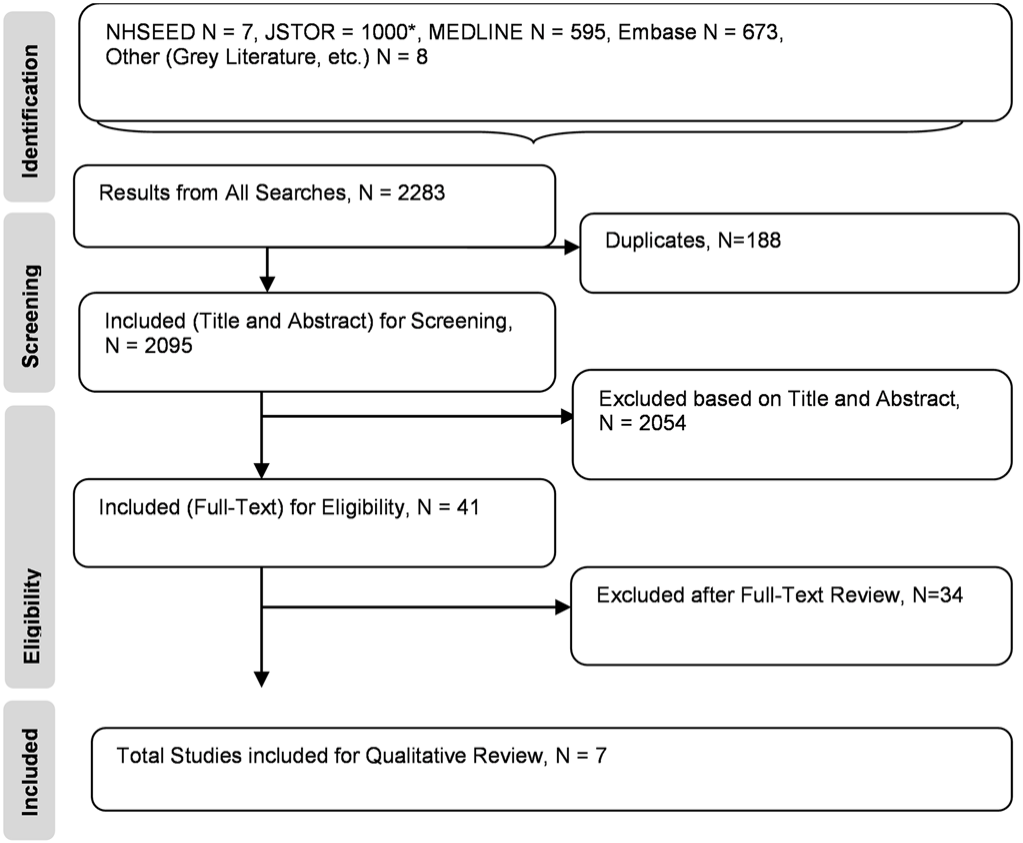
Preferred reporting items for systematic reviews and meta-analyses (PRISMA) diagram. JSTOR, Journal Storage; MEDLINE, Medical Literature Analysis and Retrieval System Online; NHSEED, National Health Service Economic Evaluation Database. 1000*: Although 1,490 articles were found using JSTOR, only 1,000 articles were accessible due to limitations of the JSTOR database.

The NHSEED, JSTOR, MEDLINE, and Embase searches yielded a total of seven articles, 1,000 articles, 595 articles, and 673 articles, respectively. An additional eight articles from the grey literature, reference lists, and referrals from subject matter experts were also included. There were a total of 2,283 studies found, and after the removal of duplicates, 2,095 were chosen for primary screening (title and abstracts). A total of 41 publications were then selected for full-text screening. Thirty-four studies were excluded after full-text review, and reasons for exclusion were recorded. (Refer to Table 1.) Seven full texts [15–21] were included for full critical appraisal and data abstraction for analysis. (Refer to Fig. 1.)

**Table 1:** Characteristics of excluded studies at second-level screening.

Author	Year	Reason excluded
Abila [44]	2007	Cost-effectiveness but interventions and outcomes related to fly population only
Brandl [45]	1988	Costs only, no effectiveness
Brightwell [46]	1991	Cost per trap discussed, paper related to effectiveness of trap as opposed to cost-effectiveness of relative comparators
Checchi [47]	2011	Screening algorithms (sensitivity/specificity outcomes only)
Esterhuizen [48]	2011	No actual costs discussed, just effectiveness of fly traps
Etchegorry [49]	2001	Costs only, no effectiveness
Fèvre [50]	2008	DALYs and burden of illness
Fèvre [51]	2008	DALYs and burden of illness
Gouteux [52]	1987	Costs only, no effectiveness
Jordan [53]	1961	Discussion only of economic importance, not actual economic analysis
Kamuanga [54]	2001	CBA using CV but outcomes based on preference for animals and not HAT
Laveissière [55]	1990	Costs only, no effectiveness
Laveissière [34]	1998	Costs only, no effectiveness
Leygues [30]	1989	Socioeconomic outcomes, not cost-effectiveness
Lutumba [56]	2005	Costs only, no effectiveness
Lutumba [57]	2006	Costs only, no effectiveness
Matemba [58]	2010	Costs and DALYs for one area, not comparative analysis
McDermott [59]	2001	Modelling of vector control only, not actual economic analysis
Mitashi [60]	2012	Screening algorithms (sensitivity/specificity outcomes only)
Mugasa [61]	2012	Screening algorithms (sensitivity/ specificity outcomes only)
Okoth [62]	1991	Costs only, no effectiveness
Putt [63]	1988	Costs only, no effectiveness
Ruiz-Postigo [64]	2001	Costs only, no effectiveness
Shaw [65]	2004	Chapter 20 about the economics of trypanosomiasis; summary of research but no formal incremental CEA
Shaw [66]	2006	Prevention and outcomes focussed on livestock, not human outcomes
Shaw [67]	2007	Costs only, no effectiveness
Shaw [68]	2009	Costs only, no effectiveness
Shaw [69]	2013	Costs only, no effectiveness
Simarro [70]	2011	Costs only, no effectiveness
Simarro [71]	2012	Costs only, no effectiveness
Trowbridge [72]	2000	Abstract only; did not mention any costs, just DALYs
Vale [73]	2005	Cost and benefits but related to vector control interventions related to fly populations only
Vos [3]	2012	DALYs and burden of illness
WHO Report [74]	1998	Costs only, no effectiveness

Abbreviations: CBA, cost-benefit analysis; CV, contingent valuation.

### Quality Assessment and Critical Appraisal

The quality scores for the seven included studies [15–21] displayed in Table 2 (SIGN Methodology Checklist 6: Economic Evaluations) demonstrated that on average 81% (67%–89%) of the items stipulated by the SIGN checklist were addressed. Economic theory suggests that individuals have a time preference in regards to gains, and hence, costs and outcomes in the future are less valuable than those in the present [22]. This concept is referred to as “discounting” and is standard methodology in economic evaluation; however, five out the seven studies in this review did not address it [16–19,21]. Each publication considered the cost and consequence compared to more than one intervention for HAT; however, three of the publications [15,17,18] did not include an incremental analysis to examine the marginal benefit of adopting one intervention compared to the next best option. A single study [19] did not have a clear objective, and Shaw’s study did not justify the study design or clearly describe the cost sources [15]. All but one study [20] completed a sensitivity analysis in addition to the base results. All studies discussed the economic importance of the question and had outcomes that could be relevant for decision makers. Overall, all studies were judged to be “acceptable” for this review.

**Table 2:** Critical appraisal (Scottish Intercollegiate Guidelines Network (SIGN) Methodology Checklist 6: Economic Evaluations).

Author	Question	Shaw [15]	Politi [16]	Shaw [17]	Lutumba[18]	Lutumba [20]	Lutumba [19]	Robays [21]
Year		1989	1995	2001	2005	2007	2007	2008
SECTION 1.	**Internal Validity**							
1.1	The study addresses an appropriate and clearly focused question	Yes	Yes	Yes	Yes	No	Yes	Yes
1.2	The economic importance of the question is clear	Yes	Yes	Yes	Yes	Yes	Yes	Yes
1.3	The choice of study design is justified	Can’t say	Yes	Yes	Yes	Yes	Yes	Yes
1.4	All costs that are relevant from the viewpoint of the study are included and are measured and valued appropriately	No	Yes	Yes	Yes	Yes	Yes	Yes
1.5	The outcome measures used to answer the study question are relevant to that purpose and are measured and valued appropriately	Yes	Yes	Yes	Yes	Yes	Yes	Yes
1.6	If discounting of future costs and outcomes is necessary, it been performed correctly	Yes	NA	No	NA	Yes	NA	Can’t say
1.7	Assumptions are made explicit and a sensitivity analysis performed	Yes	Yes	Yes	Yes	No	Yes	Yes
1.8	The decision rule is made explicit and comparisons are made on the basis of incremental analysis	No	Yes	No	No*	Yes	Yes	Yes
1.9	The results provide information of relevance to policy makers	Yes	Yes	Yes	Yes	Yes	Yes	Yes
Total fulfilment		6	8	7	7	7	8	8
		67%	89%	78%	78%	78%	89%	89%
**SECTION 2.**	**Overall Assessment of the Study**							
2.1	How well was the study conducted?	Acceptable	Acceptable	Acceptable	Acceptable	Acceptable	Acceptable	Acceptable
2.2	Are the results of this study directly applicable to the patient group targeted by this guideline?	Yes	Yes	Yes	Yes	Yes	Yes	Yes

*Base case analysis was not incremental, but sensitivity analysis had an incremental analysis

### Characteristics of Included Economic Evaluations

Each of the seven included publications had varying characteristics, as summarised in Table 3. The first publication of a full economic evaluation for HAT identified was completed in 1989 by Alexandra Shaw [15], with the next publication coming in 1995 [16]. The remaining five publications were published from 2005 to 2008 [17–21]. The evaluations covered four African countries: Democratic Republic of the Congo (DRC), Uganda, Côte d’Ivoire, and Angola. Most (3/7) evaluations (n = 3) came from DRC [18–20], with one study from Côte d’Ivoire [15], one study from Uganda [16], one study from Angola [21], and finally one study that included an analysis from both Uganda and Côte d’Ivoire [17]. Economic evaluations concerning HAT in human populations looked almost exclusively at the disease *T*. *b*. *gambiense* (71%), although in two instances the disease strain was not specified explicitly [15,21]. A total of four economic evaluations [15,18,19,21] were considered cost-effectiveness analyses (CEA) in which the cost for a desired effect or consequence (e.g., lives saved, years of infection avoided, etc.) was measured. Two studies [16,20] included both a CEA and cost utility analysis (CUA) in which the utility was measured in DALYs. One study exclusively completed a CUA in which cost per DALY averted was measured as the main outcome [17]. Overall, there was only one publication that was found in an “economic” journal, as the remaining articles were published in journals pertaining to tropical medicine and infectious diseases. Funding for the research was often not mentioned. However, WHO was referred to as a means of support in two publications [16,18], and support from the Belgian Directorate General for Development Cooperation was also mentioned [18].

**Table 3:** Characteristics of included economic evaluations.

Author	Shaw [15]	Politi [16]	Shaw [17]	Lutumba[18]	Lutumba [20]	Lutumba [19]	Robays [21]
Year	1989	1995	2001	2005	2007	2007	2008
**Type of Intervention**	Case Detection and Diagnosis + Treatment, Vector Control	Treatment	Case Detection and Diagnosis	Diagnosis	Case Detection and Diagnosis	Diagnosis	Treatment
**Country**	Côte D’Ivoire	Uganda	Uganda, Cote D’Ívoire	DRC	DRC	DRC	Angola
**Disease Strain**	Not mentioned	T. b. gambiense	T. b. gambiense	T. b. gambiense	T. b. gambiense	T. b. gambiense	T. b. gambiense*
**Type of Economic Evaluation**	CEA	CEA/CUA	CUA	CEA	CEA/CUA	CEA	CEA
**Journal**	Annales de la Société belge de médecine tropicale	Health Economics	Médicine Tropicale	Tropical Medicine and International Health	Emerging Infectious Diseases	Emerging Infectious Diseases	Tropical Medicine and International Health
**Funding**	Not mentioned	Internship at WHO	Not mentioned	WHO (Organisation mondiale de la Santé) and bourse de doctorat Direction Générale de la Coopération au Développement du Royaume de Belgique avec l’Institut de Médecine Tropicale Prince Leopold	Financed partly by doctoral grant from the Belgian Directorate General for Development Cooperation by WHO	None mentioned	None
**Additional Institutional Collaborators**	Members at WHO, member from Oxford University; VEERU	Departments in WHO: Division of Intensified Cooperation with countries, Division of Control of Tropical Diseases and Special Programme in Tropical Disease Research; Batelle MEDTAP, London; anonymous referees	TDR/WHO as Institutional collaborators	None	National Program in DRC	HAT experts	None

Abbreviations: MEDTAP, Medical Technology Assessment and Policy; TDR, Tropical Disease Research; VEERU, Veterinary Epidemiology and Economics Research Unit. *Inferred *T*. *b*. *gambiense* because of treatments being used.

### Interventions

The majority (5/7) of the publications evaluated interventions that included case detection and diagnosis, while two of the articles evaluated treatment interventions of melarsoprol and eflornithine (difluoromethlyornithine [DFMO]) for stage 2, as the treatment for stage 1 was always considered to be pentamidine [16,21]. Two publications by Lutumba [18,19] looked exclusively at sensitivity and specificity of diagnostic algorithms and staging algorithms, while one study also looked at the differences between treatment and vector control interventions in addition to case detection and diagnosis [15]. The study by Shaw in 1989 was the only publication that included a comparative economic analysis for vector control as an intervention to control HAT in a human population.

### Economic Evaluation Description

Key insights regarding the details of the included economic evaluations are described below and also summarised in Table 4.

**Table 4:** Description of included economic evaluations.

Author	Shaw [15]	Politi [16]	Shaw [17]	Lutumba[18]	Lutumba [20]	Lutumba [19]	Robays [21]
Year	1989	1995	2001	2005	2007	2007	2008
**Method/Structure**	Modelling	Modelling	Modelling	Modelling	Field Study (Economic Study)	Modelling	Modelling
**Model Description (if applicable)**	Spreadsheet model that simulates outcomes	Decision Tree with inclusion of relapses	Spreadsheet model that simulates outcomes based on the five strategies identified	**Decision Tree**	NA	Decision Tree. Complex decision tree model with separate arms for each stage of detection in the treatment algorithm specified. End diagnosis for positive tests is first or second stage of HAT. HAT-positive and HAT-negative populations examined to account Sens and Spe for TN, TP, FP, and FN.	Decision Tree. Melarsoprol and DMFO treatment arm options. Patients treated with melarsoprol have no complications or arsenic encephalopathy. Patients with no complications may relapse or be cured, while patients with an adverse event (AE) have a probability of survival prior to being cured or relapsing. Patients treated with DMFO have a probability of surviving treatment or dying. Survivors are cured or relapse. All relapse patients (DMFO and melarsoprol) have the possibility of being cured or proceed to death.
**Software**	Super-Calc 4	Not mentioned	Microsoft Excel	TreeAge	Microsoft Access, Microsoft Excel, Epi Info 2002	Data Pro 2004 (TreeAge)	TreeAge Pro 2006
**Population Description**	HAT population	1,000 hypothetical patients with *T*. *b*. *gambiense* in stage 2	100,000 hypothetical people modelled, containing 10 rural health centres and 20 community health workers	1,000,000 hypothetical patients	Economic study of 57 patients, 47 households (21%); Median age was 26 years (4–72 years); 57% of patients were female; 63% of patients in stage 1	In model 50% of patients in stage 1 and 2 equally	690 stage 2 patients
**Area Description**	Cote D’Ívoire (Vavoua focus), forest zone with scattered hamlets	Uganda	Daloa, Côte D’Ivoire/Moyo District Uganda	DRC	Single outbreak of HAT in 2000–2002 Buma, a rural community of 1,300 people (Buma centre + Kimpolo) 35 km south of Kinshasa in the DRC affected by outbreak of HAT	Probabilities, baseline data, costs and time developed from study in Kwamouth between February and March 2004	Sleeping sickness ward in Caixto, Angola
**Prevalence**	5% year one (incidence 1%)	Not mentioned	Range 0.01%–70%	1.00%	Buma: 5.92% (77 Cases/1,300 population). Based on local data: Buma centre—2% (20/1,000) Kimpolo—19% (57/300)	1.00%	Not mentioned
**Data Sources/Inputs**	CATT test & mAECT—Côte D’Ivoire	Available literature, clinical trials; reports of National Sleeping Sickness Programme-Uganda, personal communication from experts, WHO/CDT/TDR	Costs and estimates from WHO Technical Report Series 881, published in 1998	HAT Programme in the DRC, PNTHLA, literature and reports from Trypanosomiasis Bureau	Data from this study, information from PNTHLA in DRC; costs included cost consultation fees, cost of travel, lab, household expenses (except diagnostic test), and cost of hospitalization (including food for patient and caregiver); treatment costs (drug cost included but specific treatments not mentioned, injections, small materials, syringes, and needles); value of each work day lost (estimated on a person basis). DALYs were calculated estimated based on HAT-related death based on family recall and possible HAT-related deaths. Calculated HAT disability before, during, and after treatment. DALYs calculated as per Murray et al.	Annual reports from PNTHLA; study in Kwamouth, previous literature regarding Sen and Spe; treatment efficacy rates included were for first generation treatment pentamidine (stage 1) and melarsoprol (stage 2). Costs include screening, confirmation and treatment and costs generated by each algorithm. screening costs included vehicle, depreciation, operation costs, and CATT reagents.	MSF Program in Angola
**Perspective**	Not mentioned	Societal	Donors and National Healthcare System	Healthcare system	Societal	Healthcare system	Healthcare system
**Costs Valuation**	$ (USD UNK year)	$ (USD 1992)	$ (USD 1995)	$ (USD 2002)	$ (USD 2002)	€ (May 2003)	$ (USD 2005)
**Consequence Units**	1. Year of infection prevented per person.	1. DALY. 2. Life saved.	1. Patient detected. 2. DALY averted.	1. Life saved.	1. DALY. 2. Control case detected/patient cure.	1. Life saved.	1. Life saved. 2. YLL.
**Cost/Consequence valuation**	$/infection prevented	1. $/DALY averted***. 2. $/life saved.	1. $/patient detected. 2. $/DALY averted.	1. $/life saved.	1. $/DALY averted. 2. $/control case detected or patient cured.	€/life saved	1. $/life saved. 2. $/YLL averted.
**Time Horizon**	20 years (Vector Control and Screen & Treat)	NA—DT	One year (simulation repeated at different prevalence, but always same time horizon)	**NA—DT**	None	NA—DT	20 years (although this seems a bit unclear since a DT requires no discounting due to short time horizon)
**Discount rate**	10%	NA—DT	NA—one year only	NA—DT	DALYs—3%	NA—DT	10% on hospital building
**Validation**	No	No	No	No	Compared their results to other literature (e.g., Shaw and Cattand, etc.)	They discussed the limitations of the study	No
**CE Threshold**	Not mentioned	$25/DALY (World Bank)	$25/DALY (WHO)	Not mentioned	Not mentioned—just mentioned that within range of Shaw and Cattand (2001) results	Not mentioned, but competing strategies made a clear case for CE due to dominance and extended dominance	WHO-CHOICE [75] threshold; products less than GDP per capita (very cost-effective); products less than three times the GDP per capita (cost-effective)
**Alternative Scenarios/Interventions**	1. Assumption A (constant incidence): find and treat vector control (traps/targets + ground spraying). 2. Assumption B (variable incidence): find and treat vector control (traps/targets + ground spraying).	1. None. 2. Melarsoprol Melarsoprol. 3. Melarsoprol Eflornithine (DFMO). 4. Eflornithine Eflornithine (DFMO).	First Scenario: 1a. Systematic fixed postsurveillance at rural health centres (N = 1, screens 300 ppl). 1b. Road blocks near centres, usually set up on market days. 2. Filter paper sample (rural health centres N = 10, screens 3,000 ppl). 3. Filter paper sample (community health workers N = 20, screens 24,000 ppl). 4. Polyvalent mobile teams (N = 1, screens 20,000). 5. Monovalent mobile teams (N = 1, screens 36,000). Second Scenario: same as above but using data from Moyo District of Uganda	1. PG (LNP). 2. CATT. 3. PG (LNP) + CATT.	None versus active screening. 1. Treatment alone. 2. Active screening + treatment.	1. LNP-FBE-TBF. 2. LNP-CTC. 3. LNP-CATT titration-CTC-mAECT. 4. LNP-CTC-mAECT. 5. LNP-TBF-CTC-mAECT. 6. LNP-CTC-CATT titration. 7. LNP-TBF-CTC-mAECT-CATT titration.	1. Melarsoprol. 2. Eflornithine (DFMO).
**ICER Results**	Refer to Table 5.						
**Subgroup Analyses**	No	No	No	No	No	No	No
**SA**	Yes	Yes	Yes	Yes	No	Yes	Yes
**Description of SA and Results**	1. Costs were double and halved. 2. Prevalence at the start of the model. 3. Incidence in the absence of control work. 4. Stability of prevalence in the absence of control activities. 5. Number of years control was undertaken was varied. 6. Importance of animal reservoir by varying assumptions in A and B (this was a bit unclear). Results: When costs were halved or doubled, the cost per benefit unit was also halved or doubled. It was more cost-effective to carry out interventions in areas with higher prevalence. Increasing incidence made vector control more profitable under A and B, but not for finding and treating patients. Prevalence had a positive correlation with profitability over time. Adding years to which control was undertaken reduced the cost per benefit for finding and treating patients, but not for vector control. Variance in the animal reservoir had a larger impact on the cost-effectiveness of finding and treating patients than on vector control. None of these results were incremental.	1. Consequences of modified assumptions regarding treatment effectiveness. 2. Modified assumptions regarding the costs of treatments and working days lost by patients and/or relatives. 3. Other variables (under table payments, shadow price of working day, rates of noncompliance). Results: If melarsoprol is less effective than current evidence, then the relative cost-effectiveness of eflornithine would improve, making scenario/interventions “2”, “3,” and “4” potentially cost-effective. If melarsoprol effectiveness improved, then scenario/intervention “3” would be dominated by scenario/intervention “2,” making scenario/intervention “2” the most cost-effective option. If the effectiveness of eflornithine in late-stage patients is as high in refractory patients who take melarsoprol, then scenario/intervention “3” dominates scenario/intervention “4,” leaving both scenario/intervention “2” and “3” as potentially cost-effective options. Working days lost by patients and/or relatives as well as other variables had little impact on cost-effectiveness when varied.	SA looked at multiplying the number of DALYs averted per patient (which was assumed to be 15) by 1.5, 2, or 2.5 at varying prevalence. Results: Cost per DALY averted becomes more favourable as prevalence increases. None of these results were incremental.	1. The Spe of PG test was varied comparing CATT to PG + CATT. 2. Additional SA of the ($/LYS) varying the prevalence of HAT, costs of tests, and Sen/Spe of PG, CATT, and Sen of parasitology. Results: When the Spe of PG was 52%, the ICER of CATT + PG compared to CATT was $5,000/LYS. When the Spe of PG was 70%, the ICER of CATT + PG compared CATT was $3,175/LYS. When the Spe of PG was 90%, the ICER of CATT + PG compared to CATT was $1,225/LYS. Results from varying prevalence showed that $/LYS decreased as prevalence increased; however; none of these results were incremental.	NA	Looked at several parameters including prevalence of HAT, cost of mAECT, CATT whole blood Spe and Sens of CTC, mAECT, FBE, CATT whole blood, and LNP. Results: Tornado diagram demonstrated that CATT whole blood Spe had the greatest impact on the ICER; also examined function as variation of prevalence and CE ratio (but this was not an incremental analysis) was more favourable as prevalence increased. They also varied the impact of discovering the FN (data was not shown) and stated that if FNs presented themselves for treatment the differences in CE were reduced.	Authors explored both situations with drug costs and excluding drug costs. Tornado diagram demonstrated that the following parameters were examined: death rate, relapse rates of treatments, death rates and death rates due to AEs, drug costs, building costs. Results: DMFO treatment becomes CE when melarsoprol death rate is greater than 16% and when death rate due to melarsoprol is greater than 70%
**PSA**	No	No	No	No	No	No	No
**VOI**	No	No	No	No	No	No	No

** calculated ICERs based on information presented in the paper.

Abbreviations: CDT, community-directed treatment; CE, cost-effectiveness; DT, decision tree; FN, false negative; FP, false positive; FBE, fresh blood examination; NA, not applicable; PG, palpation ganglionnaire; PNLTHA, Programme National de Lutte contre la Trypanosomiase Humaine Africaine; Sen, Sensitivity; Spe, Specificity; SA, sensitivity analysis; TDR, Tropical Disease Research; TN, true negative; TP, true positive; USD, United States dollar; UNK, unknown; VOI, value of information analysis.

### Methods and Software

Six of the seven included studies used modelling to measure outcomes for the economic evaluation. Only one study completed an economic evaluation alongside a clinical trial. The most common form of modelling was decision tree modelling; the structure of the remaining models was not described in detail although they were all described as being implemented with spreadsheets. For decision tree models, TreeAge software (TreeAge Software, Williamstown, Massachusetts, United States) was used for three of four studies [18,19,21], and one publication did not mention which software was used. The two spreadsheet models that were reviewed [15,17] used Super-Calc 4 (Sorcim, Silicon Valley, California, US) and Microsoft Excel (Microsoft Corp., Redmond, Washington, US) software, while the economic evaluation alongside clinical trial (EEACT) [20] relied on Microsoft Access (Microsoft Corp., Redmond, Washington, US), Microsoft Excel (Microsoft Corp., Redmond, Washington, US), and Epi Info 2002 (Centers for Disease Control and Prevention, Atlanta, Georgia, US).

### Model Structure, Assumptions & Validation

A visual diagram of the model was provided for five of the six studies that included models [16–19,21]. Although descriptions of the six models were available, no details of the assumptions or justification for the inputs used in the modelling were addressed in any of the included literature. None of the articles reported completing an internal validation of the models, but the authors of one article [19] did compare their outcomes to other literature in similar areas for external validity.

### Population, Setting, and Perspective

In one of the modelling studies, the number of patients modelled was not mentioned, while the remaining studies included 690 to 1,000,000 hypothetical patients. The clinical trial included a total of 57 patients from 47 households [20]. As mentioned previously, the populations were based on four countries (DRC, Côte D’Ivoire, Angola, and Uganda), with different settings including: rural communities, health centres, and a sleeping-sickness hospital ward.

In one case [15], the perspective of the analysis was not mentioned, but two articles approached the economic evaluation from a societal perspective [16,20], and the remaining four articles used the provider perspective (e.g., a donor or national health service) [17–19,21].

### Additional Inputs, Outcomes, and Features of Included Economic Evaluations

Data sources for the economic evaluations came from clinical trials, primary data collection from national programmes (e.g., Programme National de Lutte contre la Trypanosomiase Humaine Africaine [PNTHLA], Médecins Sans Frontières [MSF], and National Sleeping Sickness Programme Uganda), reports from WHO, available literature, and from speaking with experts in the arena of HAT. Prevalence values were not mentioned in two studies and ranged from 0.1% to 70% in the remaining literature.

All costs were evaluated in US dollars (USD} [15–18,20,21] except for one study by Lutumba et al. [19] that estimated cost-effectiveness in euros. Three studies reported only one outcome, while the remaining studies reported two outcomes in terms of cost per outcome. Cost per DALY averted was reported in three studies, while cost per LYS was reported in four studies. Cost per years of life lost (YLL), cost per patient/control case detected or patient cured, and cost per infection prevented were also examples of cost-effectiveness reported in the literature reviewed. Shaw (1989) and Shaw and Catt and reported time horizons of 20 years and one year, respectively [15,17]. Studies that used decision tree modelling did not report time horizons as decision trees have no time-related component [16,18,19]. The two remaining studies did not report a discrete time horizon for the analysis [20,21]. Two publications reported using discount rates of 10% [15,21], while one publication reported using a discount rate of 3% [20]. The remaining publications did not mention any discounting [16–19], which was probably due to the fact that decision trees were used and therefore had no time horizon that or the time span modelled was one year or less. Two of the seven articles made explicit references to willingness-to-pay (WTP) thresholds for the cost-effectiveness of HAT as US$25/DALY [16,17]. One article mentioned that the WHO-CHOICE (CHOosing Interventions that are Cost-Effective) considered the gross domestic product (GDP) per capita of a country to be used as the WTP threshold for choosing between competing interventions [21,23]. The remaining publications [15,18–21] made no reference to a WTP threshold for the economic analysis under evaluation.

### Base Case and Sensitivity Analyses

A full description of the economic outcomes for each study is outline in Table 5. The results from the sensitivity analyses conducted for the included publications are provided in Table 4.

**Table 5:** ICER results from economic evaluations.

Author, Year	Type of Intervention	Name of Intervention	ICER Results			
			Cost/DALY Averted	Cost/LYS	Cost/YLL Averted	Cost/Control Case Detected
Shaw, 2001 [17]	Case detection and diagnosis	1. Systematic fixed postsurveillance at rural health centres	NA	NA	NA	NA
		2. Filter paper sample (rural health centres)				
		3. Filter paper sample (community health workers)				
		4. Polyvalent mobile teams				
		5. Monovalent mobile teams				
Lutumba, 2005 [18]	Diagnosis	1. CATT	-	1. -	-	-
		2. LNP		2. dominated by 1		
		3. LNP + CATT		3. $20*		
Lutumba, 2007 [19]	Diagnosis	1. LNP-FBE-TBF	-	1. -	-	-
		2. LNP-CTC		2. ED by 4		
		3. LNP-CATT titration-CTC-mAECT		3. ED by 4		
		4. LNP-CTC-mAECT		4. €76		
		5. LNP-TBF-CTC-mAECT		5. €200		
		6. LNP-CTC-CATT titration		6. dominated by 5		
		7. LNP-TBF-CTC-mAECT-CATT titration		7. €2,618		
Politi, 1995 [16]	Treatment	1. None	1. -	1. -	-	-
		2. Melarsoprol Melarsoprol	2. $8	2. $209		
		3. Melarsoprol DFMO	3. $41	3. $1,033		
		4. DFMO DFMO	4. $167	4. $4,444		
Robays, 2008 [21]	Treatment		-	*Donated drug costs not included:*	*Donated drug costs not included:*	-
		1. Melarsoprol		1. -	1. -	
		2. DFMO		2. $1596	2. $58	
				*Donated drug costs included*:	Donated drug costs included:	
				1. -	1. -	
				2.$8,169	2.$299	
Lutumba, 2007 [20]	Case detection and diagnosis, treatment	1. Treatment alone	1. -	-	-	1. -
		2. Active screening + treatment	2.$17			2. $301
Shaw 1989 [15]	Case detection and diagnosis, treatment, vector control	1. Find and Treat	NA	NA	NA	NA
		2. Vector control (traps/targets + ground spraying)				

*compared to CATT alone. Abbreviations: ED, extendedly dominated; NA, not applicable as results not reported incrementally.

A total of 5 studies [16,18–21] discussed cost-effectiveness results by calculating incremental cost-effectiveness ratios (ICERs), which are summarised in Table 5. Lutumba and colleagues published cost-effectiveness analyses of varying diagnostic algorithms for HAT [18,19]. Their results in 2005 demonstrated that lymph node puncture (LNP) in addition to CATT was more cost-effective ($20/LYS) relative to CATT alone or LNP alone [18]. In 2007, LNP followed by capillary tube centrifugation (CTC) and mini-anion exchange centrifugation technique (mAECT) (€76/LYS); LNP followed by thick blood film (TBF), CTC, and mAECT (€200/LYS); and LNP followed by TBF, CTC, mAECT, and CATT titration (€2,618/LYS) were deemed cost-effective relative to four other diagnostic algorithms. Although the strengths of these cost-effective algorithms were noted, Lutumba and colleagues noted that some of these algorithms may not be feasible to carry out in the field [19]. In regards to treatment regimens, Politi’s analysis [16] in 1995 demonstrated that based on a WTP of US$25/DALY, melarsoprol alone (initial treatment and relapses) was cost-effective at US$8/DALY (US$209/LYS) compared to no treatment. Politi’s analysis also demonstrated that a treatment pathway of melarsoprol with treatment relapses on Eflornithine (difluoro-methylornithine [DMFO]) (US$41/DALY and US$1,033/LYS) or DMFO for both treatment and relapses (US$167/DALY and US$4,444/LYS) would not have been considered cost-effective based on the aforementioned cost-effectiveness threshold of US$25/DALY [16]. A more recent publication by Robays demonstrated that DFMO was more cost-effective than melarsoprol (US$1,596/LYS and US$58/control case detected) when donated drug costs were not included; the analysis of cost-effectiveness was based on WHO-CHOICE’s suggestion that interventions at a cost of GDP per capita are very cost-effective and interventions at three times GDP per capita are cost-effective [24]. When donated drug costs were included, Robays found that DFMO was more cost-effective than melarsoprol at US$8,169/LYS and US$299/control case detected. Lutumba et al. [20] found that active screening (case detection) in addition to treatment was more cost-effective than treatment alone at $17/DALY averted and $301/control case detected or patient cured.

Two studies [15,17] did not report cost and effect results incrementally. Although Shaw et al. [15,17] conducted several analyses exploring combinations of case detection, diagnostics, treatment, and vector control, outcomes were not compared incrementally; consequently, ICERs were not attained. They did calculate $/patient detected with varying prevalence for five strategies and found that lower prevalence rates were associated with higher $/DALY and higher prevalence rates with lower $/DALY; these were based on average cost-effectiveness ratios, not ICERs.

All but one study included some form of one-way sensitivity analysis (OWSA). No studies completed subgroup analyses or conducted probabilistic sensitivity analyses (PSA), and hence, results were not presented using cost-effectiveness acceptability curves (CEAC). Additional measures of uncertainty were not explored in the form of a value of information (VOI) analysis in any of the reviewed publications.

## Discussion

A review of previous evidence has demonstrated that there have been only a few economic evaluations conducted to assess the cost-effectiveness of interventions to control HAT and reduce disease burden. From this evidence alone, it would prove difficult for decision makers to strategize on which interventions would be most cost-effective for elimination; however, the results do provide some insights into the key components of HAT disease control and how these components could be translated into HAT elimination strategies, which could then be assessed through economic evaluation.

Overall the strengths of this review are that it highlights the components that play a role in disease control and reduction of transmission and emphasizes that these are the components that should be incorporated into elimination strategies. Case detection, diagnosis, treatment, and vector control are the four categories of interventions that have been considered thus far in the literature. Strategies towards elimination should continue to consider the impact of these components but also aim to highlight their individual and collective use within a formal strategy for reaching elimination. This was highlighted in the study by Lutumba et al. [20] in which case-detection with treatment was compared to treatment alone and also in the work by Shaw and colleagues in 1989 in which essentially all four categories were evaluated with varying incidence. Within diagnostics, algorithms for CATT showed that the addition of tests led to more efficient outcomes [19]. However, there is still a gap in cost-effectiveness knowledge of the current treatment for HAT, NECT. As global investors, partners, and academic groups [10,11,25–29] are now working together not only to control and treat this disease but also to develop novel diagnostic tools [9,11] and drug treatments [10], it would be useful to compare NECT to interventions that have recently come or are near entry to the market (e.g., fexinidazole [10] and rapid diagnostic tests [9,11]). Shaw et al. [15,17] and Lutumba [20] both made reference to the benefits of combining interventions for treatment, and it would be wise for stakeholders to move beyond this and develop more complicated and time-sensitive strategies with interventions not only on their own but in combination to identify the most cost-effective pathways towards elimination.

There are still some additional considerations that have not been considered as components in HAT economic evaluations. Although *T*. *b*. *gambiense* HAT contributes to 95% of the HAT disease [5], separate strategies for *T*. *b*. *rhodesiense* could also be considered. Cultural beliefs and attitudes towards HAT will also play a role in the effectiveness of interventions [30], and although education and community sensitization programs for HAT have been evaluated in terms of their societal benefit and impact on changing knowledge and behaviour [31–33], no studies have shown their benefit in terms of cost-effectiveness. Methods of delivery and integration of health systems should also be further explored in terms of accessibility and availability, as resource constraints and lack of access in remote areas may delay elimination timelines if not considered beforehand [34,35].

### Potential Use of Cost-Effective Modelling for HAT Control and Elimination

It was quite evident from the literature review that modelling will play a role in the economic evaluation of HAT. Most of the previous economic evaluations conducted were based on models, and modelling is known to assist with forecasting future economic consequences [13]. Decision makers would benefit from the use of whole disease modelling of alternative elimination scenarios because it would allow them to consider the implications and incremental benefits of each potential strategy. Previous economic evaluation studies reliant on modelling have addressed how individual interventions reduced transmission but not how these interventions, or combinations of them, could lead to eventual elimination or interruption of disease transmission. Current modelling techniques for economic evaluation, including those used to evaluate the impact of uncertainty related to model parameters, would also be useful for decision makers in communicating the consequences of choosing non-cost-effective strategies [36]. Additionally, modelling the feasibility of interventions through health service delivery is also necessary. For example, the results from an economic evaluation regarding diagnostic algorithms [19] showed that sometimes even the most cost-effective tools may not be affordable or feasible in some of the locations where HAT occurs [19].

### Potential Use of Economic Evaluation Methodology in HAT Control and Elimination

A few considerations of cost-effective interventions could be gleaned from the few economic evaluations found. This was highlighted in the scenario described by Lutumba et al. [20] in which case-detection with treatment was more cost-effective than treatment alone, and an economic evaluation of diagnostic algorithms showed that the addition of tests to CATT could increase cost-effectiveness [19]. Treatment regimens including melarsoprol and eflornithine were considered cost-effective [16,21] for patients with HAT *T*. *b*. *gambiense*, and Politi’s analysis in 1995 also demonstrated a good understanding of economic outcomes because dominance was assessed and the importance of the efficiency frontier was illustrated [16]. Dominance refers to the economic concept that an intervention that costs less and has better outcomes relative to its comparator is considered dominant [13]. In regards to budgeting, sensitivity analyses [15,17,18] demonstrated that prevalence is related to costs. This will be important to consider because the cost per patient will increase towards the end goal of HAT elimination, but the overall cost per benefit still needs to be ascertained.

The economic evaluations reviewed presented some methodological inconsistencies. For example, there was a lack of clarity in reporting costs and consequences incrementally to a base-case scenario or relative to the next-best intervention. Historically calculations may have been done this way because of the “generalized cost-effectiveness” method [37], but if incremental and net benefits are always compared to “do nothing” instead of to the next-best option available, then the consequences of this methodology could lead to error [38]. Furthermore, when multiple strategies are being considered, dominance needs to be examined. Although four out of seven studies had more than two competing strategies, dominance was only addressed once. Evaluations that ignore dominance could lead to decision errors in which the health utility is not maximised at a societal level [13,39]. Cost-effectiveness was also referred to by the authors without making reference to a cost-effectiveness threshold. WHO-CHOICE [24] has defined thresholds previously; however, it is not clear if these thresholds values are acceptable for all global stakeholders because the authors did not always refer to a threshold value to determine cost-effectiveness.

The methodology of CEA with different interventions permits one to compare varying strategies across a disease, but the outcomes need to be unified so that decision makers can assess these comparators with ease and clarity. It is evident from this review that although CEA research may be conducted, the results are hard to interpret without standardization or reporting in a common metric (e.g., cost per DALY). Following existing guidelines for economic evaluation such as the SIGN Guidelines [14] and the more recent Consolidated Health Economic Evaluation Reporting Standards (CHEERS) statement [40] or developing guidelines that stakeholders feel acceptable for an elimination strategy would allow for consistency of analyses for HAT and other neglected tropical diseases. Formal economic evaluation guidelines and even a standard reference case have been developed by various public health funders [41–43], and researchers should consider these standards to further the future of CEA within tropical disease and disease elimination decision-making. In addition, traditional CEA measures two outcomes (cost and effects), but programs for elimination also need to consider time. Health economists will need to consider how to make recommendations to stakeholders for strategy prioritization considering all three elements for elimination.

## Conclusions

This review has demonstrated that previous research highlights the main components that play a role in elimination. Furthermore, cost-effective modelling and economic evaluation have been used and could address future economic concerns regarding elimination. Researchers interested in evaluating economic concerns regarding HAT elimination should think about modelling elimination strategies to assess cost-effectiveness using standardized methodology in order to assist stakeholder and key funders. These analyses would be of use since HAT is currently being prioritized as a NTD to reach elimination by 2020.

## Boxes

**Box 1. Key Learning Points from Economic Evaluations for HAT**

- Most interventions assessed to date to reduce and control HAT are fairly cost-effective.
- Previous publications have focused on case detection, diagnostics, drug treatments, and vector control; however, examination of combinations of interventions have not yet been assessed for HAT elimination.
- No studies to date have explored the CE of the current first-line treatment for stage one HAT, NECT.
- The feasibility of deployment of current and new interventions for HAT also should be taken into consideration in future economic evaluations.
- Previous economic evaluations demonstrate that this method can play a role in assessing the cost-effectiveness of interventions for a disease in the developing world.

**Box 2. Key Papers in Economic Evaluation of HAT Interventions**

1. Shaw AP (1989) Comparative analysis of the costs and benefits of alternative disease control strategies: vector control versus human case finding and treatment. Ann Soc Belg Med Trop 69 Suppl 1: 237–253.
2. Politi C, Carrin G, Evans D, Kuzoe FA, Cattand PD (1995) Cost-effectiveness analysis of alternative treatments of African gambiense trypanosomiasis in Uganda. Health Econ 4: 273–287.
3. Shaw AP, Cattand P (2001) Analytical tools for planning cost-effective surveillance in Gambiense sleeping sickness. Med Trop 61: 412–421.
4. Lutumba P, Robays J, Miaka C, Kande V, Simarro PP, Shaw APM, et al. (2005) [The efficiency of different detection strategies of human African trypanosomiasis by T. b. gambiense]. Trop Med Int Health 10: 347–356.
5. Lutumba P, Meheus F, Robays J, Miaka C, Kande V, Buscher P, et al. (2007) Cost-effectiveness of Algorithms for Confirmation Test of Human African Trypanosomiasis. Emerg Infect Dis 13: 1484–1490.
6. Lutumba P, Makieya E, Shaw A, Meheus F, Boelaert M (2007) Human African trypanosomiasis in a rural community, Democratic Republic of Congo. Emerg Infect Dis 13: 248–254.
7. Robays J, Raguenaud ME, Josenando T, Boelaert M (2008) Eflornithine is a cost-effective alternative to melarsoprol for the treatment of second-stage human West African trypanosomiasis in Caxito, Angola. Trop Med Int Health 13: 265–271.

## Supporting Information

S1 Supporting Information Search strategy. (DOCX)

S2 Supporting Information Inclusion-exclusion criteria legend. Abbreviations: LYG, life-years gained; HRQoL, health-related quality of life; BIA, budget impact analysis; BOI, burden of illness.
(DOCX)
