# Supplementary material for: A Literature Review of Economic Evaluations for a Neglected Tropical Disease: Human African Trypanosomiasis (“Sleeping Sickness”)
Source: PLoS Negl Trop Dis. 2015 Feb 5;9(2):e0003397. doi: 10.1371/journal.pntd.0003397 (PMC4318581; doi:10.1371/journal.pntd.0003397)
Supplement: S1 Supporting Information — (DOCX) [file pntd.0003397.s001.docx]

**Supporting Information S1. Search Strategy**

| **Type** | **Keywords for MEDLINE** | **Keywords for EMBASE** |
| --- | --- | --- |
| Population – Human African trypanosomiasis | 1. Trypanosomiasis, African/ | 1. Trypanosomiasis, African/ |
|  | 2. exp Trypanosoma brucei gambiense/ or exp Tsetse Flies/ or exp Trypanosomiasis, African/ or exp Trypanosoma brucei brucei/ or exp Trypanosoma brucei rhodesiense/ | 2. exp Trypanosoma brucei gambiense/ or exp Tsetse Flies/ or exp Trypanosomiasis, African/ or exp Trypanosoma brucei brucei/ or exp Trypanosoma brucei rhodesiense/ |
|  | 3. Trypanosom$.mp. | 3. Trypanosom$.mp. |
|  | 4. Tsetse fl$.mp. | 4. Tsetse fl$.mp. |
|  | 5. HAT.mp. | 5. HAT.mp. |
|  | 6. sleeping sickness.mp. | 6. sleeping sickness.mp. |
|  | 7. human african trypanosomiasis.mp. | 7. human african trypanosomiasis.mp. |
|  | 8. Glossin$.mp. | 8. Glossin$.mp. |
|  | 9. Tb gambiense.mp. | 9. Tb gambiense.mp. |
|  | 10. Tb rhodesiense.mp. | 10. Tb rhodesiense.mp. |
|  | 11. T brucei gambiense.mp. [mp=title, abstract, original title, name of substance word, subject heading word, keyword heading word, protocol supplementary concept, rare disease supplementary concept, unique identifier] | 11. T brucei gambiense.mp. [mp=title, abstract, original title, name of substance word, subject heading word, keyword heading word, protocol supplementary concept, rare disease supplementary concept, unique identifier] |
|  | 12. T brucei rhodesiense.mp. | 12. T brucei rhodesiense.mp. |
|  | 13. 1 or 2 or 3 or 4 or 5 or 6 or 7 or 8 or 9 or 10 or 11 or 12 | 13. 1 or 2 or 3 or 4 or 5 or 6 or 7 or 8 or 9 or 10 or 11 or 12 |
| SIGN Filter – Economic Models and Evaluations | 1. Economics/ | 1. Socioeconomics/ |
|  | 2. "costs and cost analysis"/ | 2. Cost benefit analysis/ |
|  | 3. Cost allocation/ | 3. Cost effectiveness analysis/ |
|  | 4. Cost-benefit analysis/ | 4. Cost of illness/ |
|  | 5. Cost control/ | 5. Cost control/ |
|  | 6. Cost savings/ | 6. Economic aspect/ |
|  | 7. Cost of illness/ | 7. Financial management/ |
|  | 8. Cost sharing/ | 8. Health care cost/ |
|  | 9. "deductibles and coinsurance"/ | 9. Health care financing/ |
|  | 10. Medical savings accounts/ | 10. Health economics/ |
|  | 11. Health care costs/ | 11. Hospital cost/ |
|  | 12. Direct service costs/ | 12. (fiscal or financial or finance or funding).tw. |
|  | 13. Drug costs/ | 13. Cost minimization analysis/ |
|  | 14. Employer health costs/ | 14. (cost adj estimate$).mp. |
|  | 15. Hospital costs/ | 15. (cost adj variable$).mp. |
|  | 16. Health expenditures/ | 16. (unit adj cost$).mp. |
|  | 17. Capital expenditures/ | 17. or/1-16 |
|  | 18. Value of life/ |  |
|  | 19. exp economics, hospital/ |  |
|  | 20. exp economics, medical/ |  |
|  | 21. Economics, nursing/ |  |
|  | 22. Economics, pharmaceutical/ |  |
|  | 23. exp "fees and charges"/ |  |
|  | 24. exp budgets/ |  |
|  | 25. (low adj cost).mp. |  |
|  | 26. (high adj cost).mp. |  |
|  | 27. (health?care adj cost$).mp. |  |
|  | 28. (fiscal or funding or financial or finance).tw. |  |
|  | 29. (cost adj estimate$).mp. |  |
|  | 30. (cost adj variable).mp. |  |
|  | 31. (unit adj cost$).mp. |  |
|  | 32. (economic$ or pharmacoeconomic$ or price$ or pricing).tw. |  |
|  | 33. or/1-32 |  |
