# Supplementary material for: A Literature Review of Economic Evaluations for a Neglected Tropical Disease: Human African Trypanosomiasis (“Sleeping Sickness”)
Source: PLoS Negl Trop Dis. 2015 Feb 5;9(2):e0003397. doi: 10.1371/journal.pntd.0003397 (PMC4318581; doi:10.1371/journal.pntd.0003397)
Supplement: S2 Supporting Information — Abbreviations: LYG, life-years gained; HRQoL, health-related quality of life; BIA, budget impact analysis; BOI, burden of illness. (DOCX) [file pntd.0003397.s002.docx]

**Supporting Information S2. Inclusion-Exclusion Criteria**

| **PICOS Criteria** | **Include** | **Exclude** |
| --- | --- | --- |
| Population (P) | - African trypanosomiasis in humans - Children, adults, men, women | - Chagas - American Trypanosomiasis - Other neglected diseases - Cattle and/or livestock populations - Tsetse fly populations |
| Intervention(I) & Comparators (C) | Interventions that contribute to reduction in transmission of disease in humans:   - Passive + Active Surveillance Programs - Detection and Diagnosis - Treatment - Vector Control - Other (E.g. Health systems, community sensitization, etc.) | - Interventions that DO NOT contribute to reduction in transmission of disease in humans |
| Outcomes (O) | - Costs - Consequences (E.g. $/DALYs, $/LYG, $/LYS, etc.) | - Costs only - Consequences only |
| Study (S) | - Economic evaluations as defined by Drummond et al. - CEA, CBA, CUA - Health Technology Assessment (HTA) reports with economic evaluations - Modelling studies with economic outcomes - EEACTs | - Editorials - Mathematical modeling studies with no economic outcomes - HRQoL studies - Cost analyses - BIA - BOI studies - Costing papers/studies |

CEA=Cost-effective Analysis; CUA= Cost-Utility Analysis; CBA = Cost-Benefit Analysis; DALYs =Disability adjusted Life Years, LYG =Life years gained, LYS =Life years saved , HRQoL =Health Related Quality of Life, BIA=Budget Impact Analysis, BOI=Burden of illness; EEACTs= Economic evaluations alongside clinical trials
